# Supplementary material for: An optical reaction micro-turbine
Source: Nat Commun. 2018 Oct 26;9:4476. doi: 10.1038/s41467-018-06947-y (PMC6203742; doi:10.1038/s41467-018-06947-y)
Supplement: Supplementary file 1 — Supplementary Information [file 41467_2018_6947_MOESM1_ESM.pdf]

# Supplementary Information of An optical reaction micro-turbine

S. Bianchi, G. Vizsnyiczai, S. Ferretti, C. Maggi, R. Di Leonardo

## Supplementary Note 1: Geometric characterization of microstructures

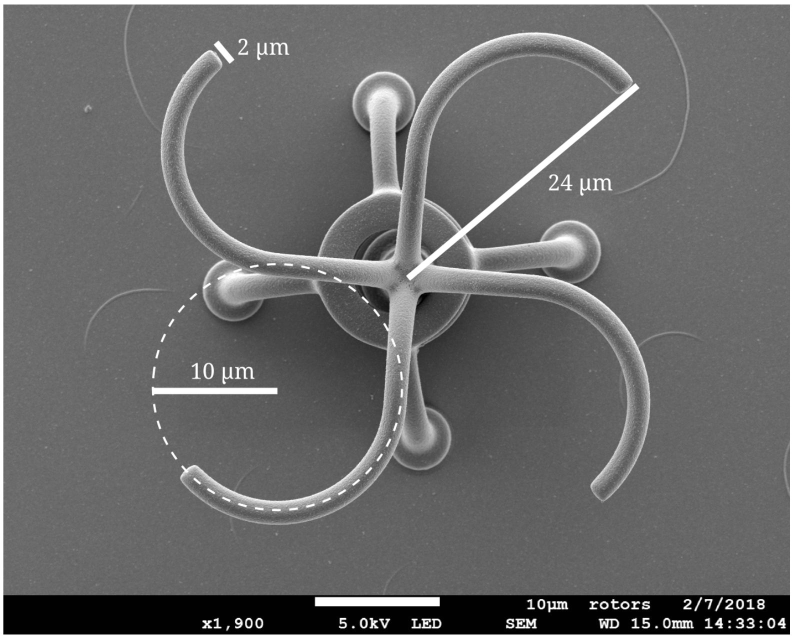


Supplementary Figure 1: Scanning electron microscopy of our optical micro-turbine. Two scalbars respectively of 2 and 24 microns are included to show the waveguide thickness and center to end distance (indicated with $r$ in the text). The dashed circle, having a radius of 10 $\mu$m, highlights the curvature of the arm.

Supplementary Fig. 1 shows a scanning electron microscopy image of a micro-turbine. Distance between the central core and the end of an arm, which is 24.1 $\mu$m by design, is compared with a scale bar of 24 $\mu$. Another scalebar of 2 $\mu$ is also reported to show the agreement with the desired waveguide thickness. Finally the dashed circle shows the radius of curvature of the arm (10 $\mu$m in the design) .

## Supplementary Note 2: Experimental determination of drag coefficients

In addition to our four arm structures we fabricated two other variants with four and three arms. A bright-field images of the structures with two, three, and four arms are shown in Supplementary Fig. 2(a-c).

To measure the rotational drag of the micro-structures we tracked its Brownian fluctuations. The tracking is based on a shape recognition algorithm having as a input a synthetic image computed by projecting the 3d model of the rotor on the focal plane. At each frame such a model image is rotated and shifted until the overlap with the experimental digital image is maximized. In this way we can track precisely the center and the orientation angle of the rotor.

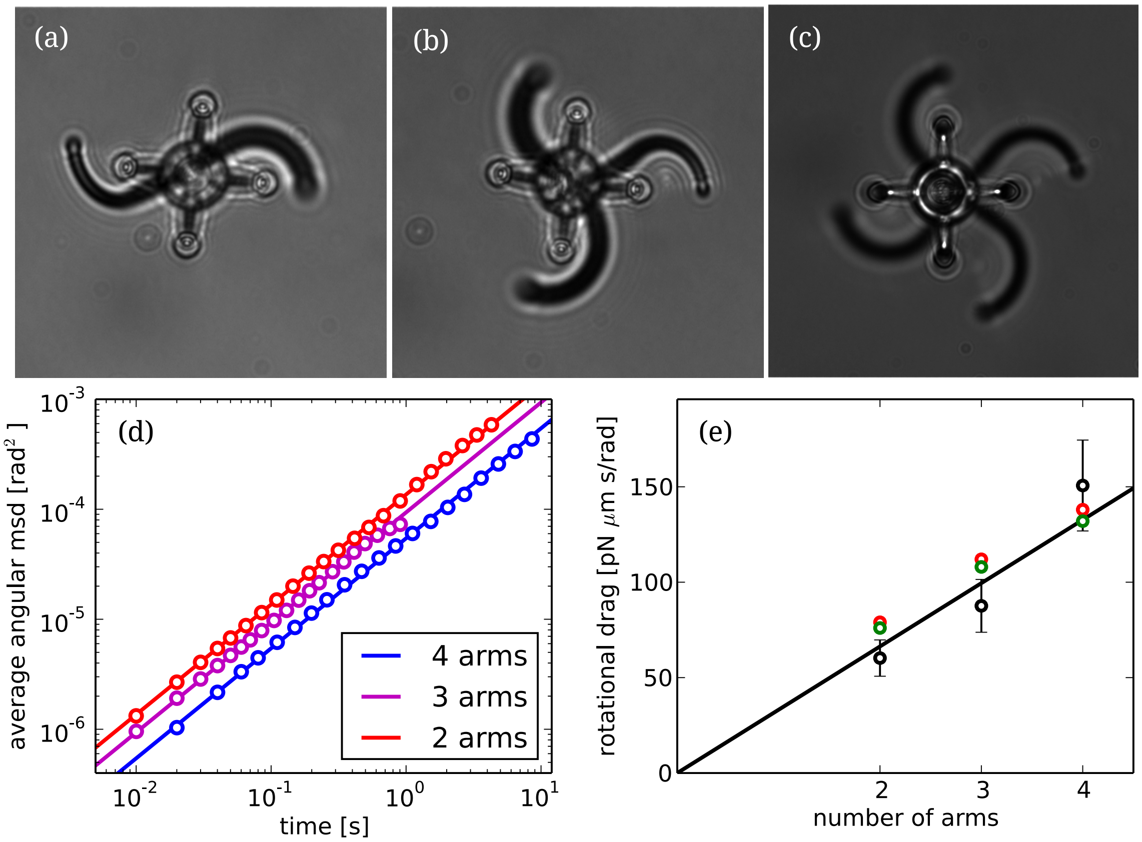


Supplementary Figure 2: Bright-field images the of our optical turbines in which the waveguide splits in two (a), three (b), and four (c) arms. (d) Angular mean square displacements of the three rotors shown in (a-c). (e) Drag coefficients of the rotors. Black circle plot experimental data. Error bars represents the standard error of the mean drag coefficients obtained from four independent measurements. Red and green circles plot values obtained numerically with a Rotne-Prager method. Green circles indicate the drag of the rotor in the bulk while red circles indicate the drag of the rotor when stator unit and coverglass is also present (see Supplementary Fig. 3).

From the angular coordinate obtained with the tracking we compute the mean square displacement (MSD). Empty circles in Supplementary Fig. 2(d) plot the MSDs of the structures. The MSDs can be fitted to the function:

$$MSD(t) = 2D_r t \tag{1}$$

where $D_r$ is the angular diffusion coefficient. Fits to experimental data are plotted in Supplementary Fig. 2(d) by solid lines. Finally, using Stokes-Einstein relation, we obtain the drag $\gamma = k_B T/D_r$ (where $k_B$ is the Boltzmann constant and $T$ is the absolute temperature). The drag of the three structures are plotted in Supplementary Fig. 2(e); the plot shows that, within the errors, the drag increases with the number of arms suggesting that the friction due to proximity of the stator and the rotor is negligible and most of the drag is due to the arms.

At rest the structures lay on the stator however, when a laser beam is coupled into the waveguide, the rotor tend to align vertically and rotate smoothly without touching the stator. While the rotation of the four-armed structure is stable, rotors with two and three arms, after a few second of rotation, adhere to the stator even in deionized water. When rotation is stopped by adhesion it can be restarted with a small external shock obtained by gently touching the microscope stage.

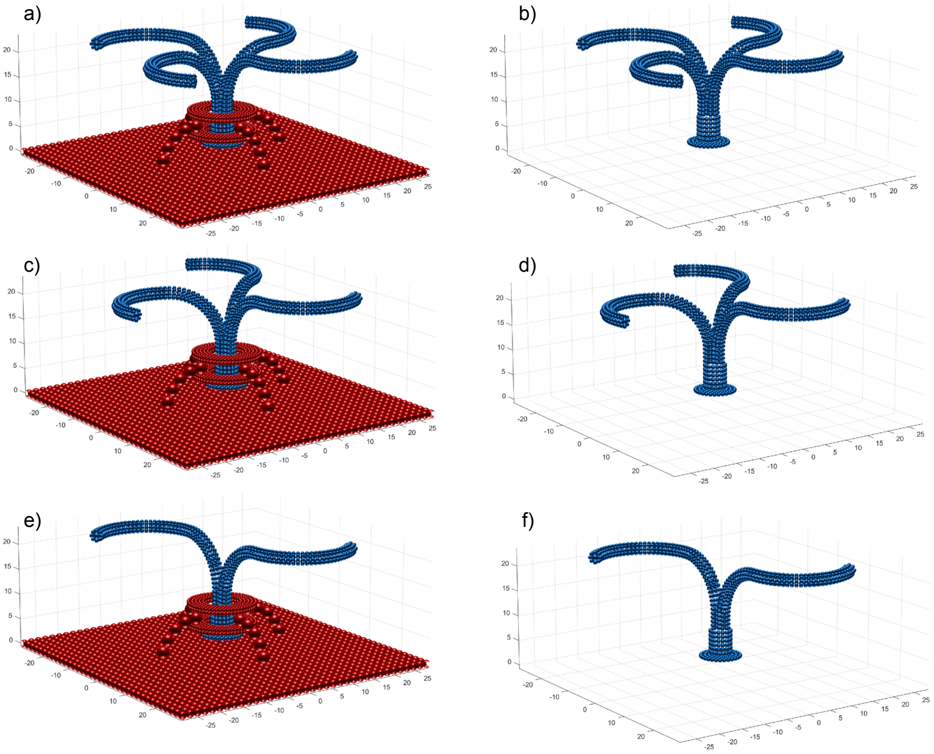


Supplementary Figure 3: Microstructures are discretized as assemblies of spherical microbeads of different radii for drag calculation with a Rotne-Prager method [1]. For each arm number we considered both the case of complete structures (left column) and rotor only (right column).

# Supplementary Note 3: Numerical calculation of drag coefficients

Experimental values for the rotational drag obtained in the previous section have been compared with numerical calculations obtained using a Rotne-Prager method [1]. We considered the cases of 2, 3, and 4 armed rotors both in the presence of stator unit and coverglass and in the bulk (see Supplementary Fig. 3). Numerical simulations indicate that the presence of the stator unit and of the coverglass affect drag only slightly. All values are reported in Supplementary Fig.2(e) evidencing a satisfactory agreement with experimental values.

# Supplementary References

[1] Reichert Michael, *Hydrodynamic interactions in colloidal and biological systems*, University of Constance Constance, Germany (2006).
