# Supplementary material for: An optical reaction micro-turbine
Source: Nat Commun. 2018 Oct 26;9:4476. doi: 10.1038/s41467-018-06947-y (PMC6203742; doi:10.1038/s41467-018-06947-y)
Supplement: Supplementary file 2 — Description of Additional Supplementary Files [file 41467_2018_6947_MOESM2_ESM.pdf]

Description of Additional Supplementary Files

File Name: Supplementary Video 1
Description: Optical microscopy movie of a rotating micro-turbine
